# Trajectories of Drug Use and Depressive Symptoms among Latinx Youth and Sexual Minority Youth

**DOI:** 10.3390/ijerph20105883

**Published:** 2023-05-19

**Authors:** Alyssa Lozano, Tae Kyoung Lee, Elliott R. Weinstein, Yannine Estrada, Beck Graefe, Maria I. Tapia, Guillermo Prado

**Affiliations:** 1 Department of Public Health Sciences, Miller School of Medicine, University of Miami, Miami, FL 33136, USA; adl122@miami.edu; 2 Department of Child Psychology and Education/Social Innovation Convergence Program, Sungkyunkwan University, Seoul 03063, Republic of Korea; ltk501@skku.edu; 3 Department of Psychology, University of Miami, Coral Gables, FL 33146, USA; erw73@miami.edu; 4 School of Nursing and Health Studies, University of Miami, Coral Gables, FL 33146, USA; yestrada@miami.edu (Y.E.);; 5 Department of Educational and Psychological Studies, University of Miami, Coral Gables, FL 33146, USA

**Keywords:** depressive symptoms, drug use, Latinx, sexual minority youth, latent class analysis

## Abstract

Drug use and depression co-occur and disproportionately affect Latinx sexual minority youth relative to their heterosexual Latinx peers. However, heterogeneity in co-occurring patterns of drug use and depressive symptoms is unknown. The objective of the current study was to identify patterns of drug use and depressive symptom trajectories and examine how these patterns varied between Latinx sexual minority youth and Latinx non-sexual minority youth. Latent class trajectory analysis identified distinct patterns of drug use and depressive symptom trajectories among 231 Latinx adolescents (Latinx sexual minority youth: *n* = 46, 21.4%; Latinx non-sexual minority youth: *n* = 169, 78.6%). After identifying class mean trajectories, we examined differences in mean trajectories across groups. A 3-class model was selected as the optimal class trajectory model for both groups, yet classes and trajectories differed. There were differences in initial levels of depression and drug use trajectories between both groups, as well as differences in patterns of drug use trajectories between both groups in two of the three classes. Given the variation in trajectory patterns, there is a need for practitioners to consider the unique needs of both groups to inform the development of preventive interventions for these two populations.

## 1. Introduction

Depressive symptoms and drug use typically develop during adolescence and are often comorbid among adolescents who develop either of these outcomes [1,2]. For example, drug use behaviors (i.e., marijuana, opioid, risky alcohol, and cigarette use), are more prevalent among adolescents who reported a past-year major depressive episode than adolescents without a past-year major depressive episode [3]. Compared with adolescents with either depression or drug use alone, adolescents with both conditions are at higher risk for self-injury, academic failure, violence, and suicide [2,4].

Studies suggest that some adolescent subpopulations, such as sexual minority youth (SMY; e.g., gay, lesbian, or bisexual youth), experience a disproportionate disease burden and subsequently face significant general and behavioral health disparities [5]. Relative to heterosexual youth, SMY report significantly higher rates of depressive symptoms [6] and drug use [7]. Both outcomes often emerge in adolescence, a formative developmental period when many individuals develop mental health conditions and risk behaviors such as drug use. Moreover, these outcomes tend to co-occur and peak with each other [8]. 

Challenges related to navigating the development of racial and ethnic identities and the associated expectations of their respective ethnicities may potentially contribute to Latinx sexual minority youth (LSMY) facing disproportionately negative health outcomes such as depressive symptoms [9] and drug use [10] relative to their non-Latinx, heterosexual, and sexual minority peers. For example, in a quantitative study by Richter and colleagues [11], Latinx parents were found to have more negative attitudes about gay or lesbian sexuality relative to non-Latinx parents, which may be influenced by constructs such as *familismo* and *machismo*, cultural values that are related to traditional family values and gender roles, respectively [12]. Accordingly, Latinx youth report higher rates of negative reactions upon disclosure of their sexual orientation relative to non-Latinx whites [13]. Family rejection of a youth’s orientation during the coming-out process is a significant predictor of depression [14] and drug use [13]. Moreover, because formation of a sexual identity is a critical developmental process that begins in adolescence and may subsequently change over time, we would expect that as sexual identity changes, there may be changes in patterns of depression and drug use for LSMY [15,16]. Although Latinx non-SMY do not have to navigate their sexual identities in terms of being a sexual minority, they are vulnerable to peer influences, which may consequently impact drug use and depressive symptoms.

Despite the disproportionate impact of comorbid depressive symptoms and drug use among LSMY, few studies [17] have examined how this co-occurrence of depressive symptoms and drug use may vary among SMY relative to non-SMY. Similarly, past research has predominantly used cross-sectional data, which limits understanding of longitudinal changes in drug use and depression across adolescence. Furthermore, to our knowledge, no studies have examined these co-occurring patterns of depressive symptoms and drug use exclusively with a sample of LSMY. Therefore, the main purpose of the current study was to (1) identify distinct patterns of drug use and depressive symptom trajectories across adolescence and (2) examine how identified trajectory patterns vary among LSMY and Latinx non-SMY. 

## 2. Materials and Methods

This secondary data analysis utilized data from the control arm of an ongoing randomized controlled trial evaluating the relative effectiveness of an online parenting intervention in preventing adverse health outcomes among Latinx adolescents ages 12–17 (Clinical Trials Identifier: NCT03009539). Further details are provided elsewhere [18]. However, adolescents were screened for eligibility via a brief survey that verified the inclusion criteria. Adolescents were eligible to participate if they: (1) were of Hispanic immigrant origin, defined by at least one parent born in a Spanish-speaking country of the Americas; (2) were between the ages of 12 and 17 years; (3) were living with an adult primary caregiver who was willing to participate; and (4) had broadband Internet access on a device. The study was approved by the University of Miami Social and Behavioral Sciences Institutional Review Board. Parental consent and adolescent assent were obtained for all participants.

### 2.1. Participants and Procedures 

The present study sample consisted of 231 Latinx adolescents in South Florida (LSMY: *n* = 46, 21.4%, and Latinx non-SMY: *n* = 169, 78.6%). LSMY were identified based on their self-reported responses to the Klein Sexual Orientation Grid to questions related to their identity, attraction, behavior, emotional connection, and fantasies toward either the same or both sexes [19]. Some adolescents did not answer any of these questions (*n* = 16), therefore they were excluded from the analysis. 

### 2.2. Measures

Study measures evaluated depressive symptoms, frequency of past 90-day drug use, sociodemographic characteristics, and family functioning. Study data were collected online, under the supervision of a study assessor, and managed using REDCap electronic data capture tools [20] at four timepoints: baseline, 6-, 12-, and 24-months post-baseline. All reported Cronbach’s alphas below are from the present study sample. Although these measures have not been formally validated with the present study sample, they have been used in previous trials with Latinx adolescents [21,22]. 

The 20-item Center for Epidemiologic Studies Depression Scale (α = 0.89) [23] was utilized to measure average depressive symptoms among adolescents. Respondents were asked to report how often they felt symptoms of depression in the past week. For example, “I felt that I could not shake off the blues even with help from my family and friends.” Response choices ranged from “1 = rarely or none of the time (less than 1 day)” to “4 = all of the time (5–7 days)”. 

Items from the Monitoring the Future survey [24] were adapted to assess the past 90-day adolescent drug use frequency of illicit drugs (i.e., marijuana, inhalants, cocaine, LSD, PCP, ecstasy, mushrooms, speed, ice, and heroin). For example, “On how many occasions have you smoked marijuana, used inhalants, cocaine, LSD, PCP, ecstasy, mushrooms, speed, ice, or heroin in the past 3 months?” Past 90-day frequency items for marijuana, inhalants, cocaine, LSD, PCP, ecstasy, mushrooms, speed, ice, and heroin were combined (i.e., endorsements were summed) into one past 90-day drug use variable. 

To understand the context of co-occurring depressive symptoms and drug use, adolescent age, gender, nativity (i.e., U.S. born or foreign-born), and time in the U.S. were included as covariates in the model. Parent nativity (i.e., U.S.-born or foreign-born), time in the U.S., education level, and income were also examined. Measures of family functioning were also used as covariates. These measures pertain to parent-adolescent communication [25], parental monitoring of peers [26], positive parenting and parental involvement [27], and family communication [28]. Parent-adolescent communication (Cronbach’s α = 0.88; 20 items assessed communication between parents and adolescents; for example, “My primary caregiver tries to understand my point of view.” Parental monitoring of peers (Cronbach’s α = 0.80; 6 items) asked the extent to which parents monitor adolescents’ activities and friends with questions such as: “How well do your parents know your best friends?**”** The Parenting Practices Scale assessed both positive parenting (Cronbach’s α = 0.86; 9 items) and parental involvement (Cronbach’s α = 0.85; 15 items). Positive parenting examined how well parents positively reinforce adolescents; for example, “When you have done something that your primary caregiver likes or approves of, how often does your primary caregiver give you a wink or a smile?” and parental involvement assessed how frequently adolescents and parents did activities together, “How often do you and your primary caregiver do things together at home?” Finally, family communication (Cronbach’s α = 0.71; 3 items) examined communication between family members: “My family knows what I mean when I say something.” Higher scores on these subscales indicate greater communication, monitoring, positive parenting, parental involvement, and family communication, respectively. 

### 2.3. Data Analytic Plan

First, means and standard deviations for continuous variables and proportions for categorical variables were investigated across all study variables (i.e., depressive symptomology and frequency of past 90-day drug use) and sociodemographic characteristics (Table 1). Second, latent class trajectory analysis [29], a latent variable modeling technique that identifies unobserved subgroups of individuals within a population, was used to identify heterogeneous groups (latent classes) of adolescents based on their trajectories (patterns) of depressive symptoms and drug use at baseline, 6-, 12-, and 24-months post-baseline. 

To identify heterogeneous groups of adolescents based on patterns of depressive symptoms and drug use, we fit the data to a series of class models with increasing numbers of latent classes. For example, we examined the data to determine if the pattern of depressive symptoms and drug use could be best grouped into 2, 3, or 4 categories (i.e., class models). Drug use class trajectories were estimated based on the Poisson distribution because the past 90-day drug use was treated as a zero-inflated count variable. Models were assessed using different information criteria to identify the model that best fits the data. We considered the Bayesian Information Criterion (BIC) and the sample-size-adjusted Bayesian Information Criterion [SSBIC], with lower values preferred [30]. The Lo Mendell-Rubin likelihood ratio tests (LMR-LRT) were also used as criteria; a significant *p*-value of LMR-LRT indicates that the estimated k class has a better fit compared to the class below (i.e., the k-1 class) [31]. Finally, we looked for higher values of entropy when comparing classes, which tells us how accurately the model defines the classes [32]. In addition to fit statistics, class interpretability and class size (i.e., class size *n* > 10%) were also considered in the model enumeration (i.e., the class model decision) process [33]. 

After identifying the optimal class model, mixture modeling with known class membership [34,35] was utilized to compare the identified class mean trajectories between known classes of sexual orientation (i.e., LSMY and Latinx non-SMY). Using Wald chi-square tests, differences in mean trajectories of depressive symptoms and drug use between LSMY and Latinx non-SMY were examined within each class using MPlus v8.3 [36]. 

Missing data were accounted for using Full Information Maximum Likelihood (FIML) procedures [37]. The average proportion of missing data for the depressive symptom variable was 12.5% and 12.8% for the drug use variable. Finally, class memberships were exported to SPSS [38] to conduct a 3-step approach [39] to estimate the relationship between the separated classes and study covariates (i.e., sociodemographic characteristics and family functioning). In this approach, the measurement parameters of the latent classes are held fixed, and then auxiliary variables (i.e., covariates) are subsequently included and their relation to the latent class variable is estimated [40]. One-way analysis of variance (ANOVA) or chi-square tests were conducted to assess these relationships, and *p*-values less than 0.05 were considered significant. 

## 3. Results

The mean adolescent age was 13.84 years (SD = 1.38). A slight majority of adolescents were female (51.5%) and born in the United States (55.4%). Table 1 outlines all baseline participant demographic information.

### 3.1. Latent Class Trajectories for Overall Sample

**Figure 1:**
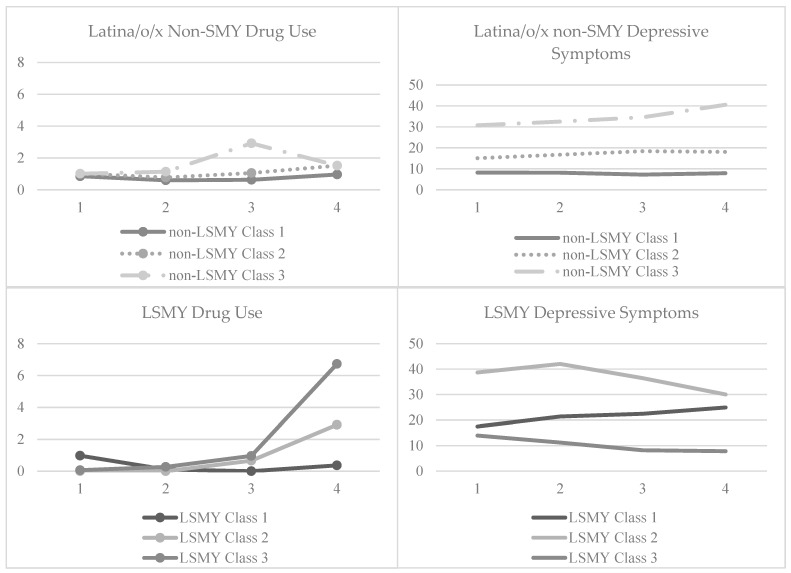
Plots for Latinx non-SMY and LSMY samples. Latinx non-LSMY classes: Class 1: low initial depressive symptoms and drug use that are both stable over time; Class 2: moderate initial depressive symptoms and low initial drug use that are both stable over time; and Class 3: high initial depressive symptoms that increase over time and low initial drug use that increases then decreases over time. LSMY classes: Class 1: moderate initial depressive symptoms that increase over time and low initial drug use that decreases over time; Class 2: high initial depressive symptoms that decrease over time and low initial drug use that increases over time; and Class 3: sub-clinical depressive symptoms that increase over time and low initial drug use that increases over time.

A three-class solution was identified for the overall sample based on information criteria (IC), delineation of classes, class size, and interpretability (Table 2). Similarly, a three-class model was selected as the optimal class trajectory model for both Latinx non-SMY and LSMY samples. Although IC statistics (i.e., BIC and SSBIC) were lower for a four-class solution and the adjusted LMR-LRT was significant, there were concerns with that class solution due to significant conceptual overlap among some of the classes in those solutions and small sample sizes of some of the classes (i.e., less than 5% or less than 10 individuals in the classes, Table 2). Further, although the LMR-LRT was significant for the Latinx non-SMY two-class solution, other fit indices (i.e., BIC and entropy) indicated that a three-class solution was a better fit overall. For the overall sample, the classes were (a) low depressive symptoms that are stable over time [Estimated means of initial level (I) = 8.30, *p* < 0.001, Estimated means of slope level (S) = 0.03, *p* = 0.92] with high drug use that decreases over time (I = 3.87, *p* < 0.001 S = −0.14, *p* < 0.05; *n* = 125, 54.3%), (b) high depressive symptoms that are stable over time (I = 34.28, *p* < 0.001, S = −0.70, *p* = 0.56) with low initial drug use that increases over time (I = 0.48, *p* = 0.45, S = 0.95, *p* < 0.001; *n* = 23, 10.0%), and (c) moderate depressive symptoms (I = 17.46, *p* < 0.001, S = 0.49, *p* = 0.33) with moderate drug use that increases over time (I = 1.28, *p* < 0.001, S = 0.26, *p* < 0.02; *n* = 82, 35.6%). Patterns of three class trajectories for Latinx non-SMY and LSMY are shown in Figure 1; classes were labeled based on the magnitude of the slope, irrespective of the significance.

### 3.2. Latent Class Trajectories for Latinx Non-SMY

As shown in Figure 1, for Latinx non-SMY, patterns of three class trajectories were: (a) low initial depressive symptoms (I = 8.06, *p* < 0.001, S = −0.10, *p* = 0.73) and drug use that are both stable over time (I = 3.87, *p* < 0.001, S = −0.14, *p* < 0.05; *n* = 99, 58.6%; class 1), (b) moderate initial depressive symptoms (I = 15.80, *p* < 0.001, S = 0.75, *p* = 0.11) and low initial drug use (I = 1.19, *p* < 0.001, S = 0.25, *p* = 0.15; *n* = 60, 35.5%; class 2) that are both stable over time, and (c) high initial depressive symptoms that increase over time (I = 30.21, *p* < 0.001, S = 2.44, *p* = 0.13) and low initial drug use that increases then decreases over time (I = −1.73, *p* < 0.05, S = 2.37, *p* < 0.001; *n* = 10, 5.9%; class 3).

### 3.3. Latent Class Trajectories for LSMY

As shown in Figure 1, for LSMY, patterns of three class trajectories were: (a) moderate initial depressive symptoms that increase over time (I = 18.75, *p* < 0.001, S = 1.69, *p* = 0.23) and low drug use that decreases over time (I = 1.59, *p* < 0.001, S = −0.12, *p* = 0.58; *n* = 21, 45.6%; class 1), (b) high initial depressive symptoms that decrease over time (I = 41.29, *p* < 0.001 S = −2.59, *p* < 0.01) and low initial drug use that increases over time (I = −0.82, *p* = 0.18, S = 0.97, *p* < 0.001; *n* = 10, 21.7%; class 2), and (c) sub-clinical depressive symptoms that increase over time (I = 12.51, *p* < 0.001, S = −1.43, *p* < 0.05) and low initial drug use that increases over time (I = 0.41, *p* < 0.001, S = 1.06, *p* < 0.001; *n* = 15, 32.6%; class 3).

### 3.4. Differences in Class Trajectories between Latinx Non-SMY and LSMY

Table 3 presents the results of the known class analysis model parameter tests. There were significant differences in initial levels of depression and drug use between LSMY and Latinx non-SMY in all classes except for class 2 (moderate depression and moderate drug use that increases over time) for drug use (Table 3). There were also significant differences in the patterns of the slope of drug use experienced by LSMY and Latinx non-SMY in classes 1 (high depression with increasing drug use over time; χ^2^ = 33.78, *p* < 0.001) and 3 (low depression with high initial drug use that decreases over time; χ^2^ = 440.25, *p* < 0.001).

### 3.5. Relationship between Identified Latent Classes and Study Covariates

Table 4 presents covariate effects by class membership. There was a significant relationship between all the separated classes and gender [χ^2^ (5) = 27.27, *p* < 0.001, ϕ = 0.36] and adolescent-reported parent-adolescent communication [F(5, 197) = 9.22, *p* < 0.001, η^2^ = 0.19; Table 4]. See Supplemental Table S1 for Tukey’s HSD Test for multiple comparisons for parent-adolescent communication. Notably, parent-adolescent communication was significantly higher in Latinx non-SMY class 3 than in LSMY classes 1 and 2.

## 4. Discussion

We identified three distinct trajectory patterns of co-occurring drug use and depressive symptoms for LSMY and Latinx non-SMY. Latent classes for Latinx non-SMY included: (1) low initial depressive symptoms and low initial drug use that are both stable over time; (2) moderate initial depressive symptoms and low initial drug use that are both stable over time; and (3) high initial depressive symptoms that increase over time and low initial drug use that increases then decreases over time. Latent classes for LSMY included: (1) moderate initial depressive symptoms that increase over time and low initial drug use that decreases over time; (2) high initial depressive symptoms that decrease over time and low initial drug use that increases over time; and (3) sub-clinical depressive symptoms that increase over time and low initial drug use that increases over time.

Study results also indicate that the three identified class trajectory patterns of drug use and depression varied between LSMY and Latinx non-SMY adolescents. Patterns in baseline levels and slopes were significantly different between the groups. Apart from class 2, baseline levels of depressive symptoms and drug use are different across all classes and groups, suggesting that for classes 1 and 3 in both groups, the baseline level of depressive symptoms does not significantly change over time. However, the pattern of depressive symptoms over time was statistically significant in class 2, suggesting that LSMY and Latinx non-SMY have different patterns of depressive symptoms over time. For drug use, in classes 1 (high depression with increasing drug use over time) and 3 (low depression with high initial drug use that decreases over time), there were significant differences in the trajectory of drug use over time across classes in both groups. 

By using a person-centered approach (i.e., latent class analysis), we were able to describe the heterogeneity in populations based on multiple characteristics (i.e., depression and drug use), which provides a deeper understanding of the co-occurrence of these outcomes. Reasons for different patterns of depressive symptoms and drug use among LSMY and Latinx non-SMY may be attributed to varying societal expectations and/or pressures for both groups. LSMY must navigate their intersecting identities—both sexual orientation and racial and ethnic—and the associated expectations of Latinx culture. For example, many LSMY may choose not to disclose their sexual orientation to their families to avoid disrupting family harmony (i.e., familismo) due to perceived negative reactions from family members [41]. This decision may subsequently lead to an increased likelihood of poorer behavioral health (i.e., depressive symptoms and drug use) due to the burden of withholding information from one’s own family. Further, if LSMY decide to disclose their sexual orientation and their parents have a negative reaction to the disclosure, negative reactions may be a significant predictor of subsequent depression and illicit drug use [13,14]. 

Lack of parent acceptance of youths’ sexual orientation may have been reflected in our sample given that parent-adolescent communication was significantly lower for LSMY in classes 1 and 2 relative to Latinx non-SMY in class 3. It is possible that poor parent-adolescent communication may have resulted from family rejection of LSMY’s sexual orientation, which follows the patterns of increasing depressive symptoms with moderate drug use (LSMY class 1) and increasing drug use (LSMY class 2). Further, initial levels of drug use for LSMY were consistently moderate or high and remained moderate or increased over time, and baseline levels of depressive symptoms were either moderate, high, or increasing over time. These patterns may have been related to family processes related to parent support/rejection and, consequently, parent-adolescent communication. Moreover, the LSMY group contained more females than the Latinx non-SMY group, and the classes within the LSMY groups showed increasing, moderate, or high levels of depression. In fact, LSMY classes 1 and 2 were composed entirely of females. Data indicate that sexual minority females have significantly higher levels of depressive symptoms relative to sexual minority males and non-sexual minority males and females [42], which may have impacted the patterns of the LSMY classes with female LSMY and also potentially driven the differences in levels of depressive symptoms between LSMY and Latinx non-SMY, such that the LSMY subgroup had a statistically significant higher average of depressive symptoms relative to Latinx non-SMY. Public health practitioners should be cognizant of the unique needs of male and female LSMY, such that female LSMY may require additional scaffolding to manage depressive symptoms.

Similarly, Latinx non-SMY also had varying patterns of co-occurring depressive symptoms and drug use. This is not surprising given that Latinx non-SMY also face several challenges during adolescence. For example, adolescence is also a time when the influence of peers becomes increasingly important. Adolescents may seek approval from their peers, which in turn makes them vulnerable to peer influence [43,44], a proximal risk factor for adolescent drug use [45]. Additionally, if adolescents do not succumb to peer influence or pressure, they may face rejection by their peers and develop depressive symptoms [46,47]. For adolescents (i.e., Latinx non-SMY class 3) who associate with antisocial peers, parental monitoring may be lacking. Both factors may potentially contribute to high depressive symptoms and unfavorable drug use patterns. 

Notably, however, both groups appeared to have made improvements in some outcomes. For example, for Latinx non-SMY in class 3, drug use decreased over time. It may be that these are impulsive youth who are experimenting with drugs. Similarly, for LSMY in classes 2 and 3, depressive symptoms decreased over time. Considering that 36% of SMY have received mental health care [48], it may be that youth in this class have received some kind of mental health treatment and/or have strong social or family supports that have helped them navigate their depressive symptoms. 

Although Latinx non-SMY and LSMY both face the typical challenges associated with adolescence, each group has varying patterns and trajectories of co-occurring depressive symptoms and drug use. Given this variation, there is a need for targeted preventive interventions so that public health practitioners can address the unique needs of both groups. Family-based interventions may be an efficient mechanism to target poor behavioral health (i.e., depressive symptoms and drug use), given that family-based interventions originally developed to address drug abuse prevention have also shown crossover effects in reducing the prevalence of mental health symptoms among Latinx adolescents [49]. 

However, despite the efficacy of family-based interventions in preventing or reducing such outcomes, they may not function in the same way for LSMY. A study by Ocasio et al. [50] that examined the relative efficacy and effectiveness of a general family-based intervention on risk behavior outcomes in LSMY found that the hypothesized family functioning mediators of the intervention did not mediate intervention effects on risk outcomes for LSMY. It may be that there are LSMY-specific minority stressors that exist within the family and with peers that need to be resolved to maximize improvements in general family functioning processes. Additionally, as suggested by our findings, parent-adolescent communication may not be as strong for LSMY due to the possible ramifications of parental rejection of the adolescents’ sexual orientation. 

This suggests that there is a need to build on the existing literature to both adapt and enhance existing evidence-based family interventions for LSMY by focusing on diminished parent-adolescent communication as a means of improving depressive symptoms and problematic patterns of drug use [50]. Such interventions should also deliver LSMY-relevant information to both LSMY and their parents by focusing on the unique circumstances these families are experiencing (e.g., accepting their adolescent’s sexual orientation) [51] in order to appropriately target the unique needs of LSMY’s behavioral health. Therefore, public health practitioners should assess family-level dynamics (e.g., acceptance, parent-adolescent communication) when engaging LSMY and their families to help inform the etiology of LSMY’s depressive symptoms and/or drug use. Furthermore, practitioners should meet families where they are with regard to processing the disclosure of youths’ sexual orientation. 

Similarly, for Latinx non-SMY, family-based interventions that focus on family processes such as parental monitoring of peers may ameliorate the pressure felt by adolescents to use drugs and protect against depressive symptoms [49]. By instilling skills in parents to effectively monitor their adolescents, parents can be aware of what adolescents are doing and with whom and take steps to protect youth from associating themselves with other antisocial peers who may negatively impact adolescents’ substance use. With simultaneous improvements in parent-adolescent communication, parents can develop the appropriate skills to have conversations related to monitoring their adolescents’ activities while also fostering a unique form of social support for adolescents to counteract possible depressive symptoms they may be facing due to peer rejection or other pressures of adolescence. In addition to providing parents with skills, public health practitioners should assess youths’ relationships with their peers to understand whether depressive symptoms and/or drug use are related to peer influence. Further, examining the patterns of youths’ drug use (e.g., consistent use, experimentation only) can inform unique approaches to addressing drug use and evaluate how depressive symptoms may result from or exacerbate drug use. 

Future studies that examine co-occurring trajectories of drug use and depressive symptoms should ascertain which began first. Understanding whether drug use occurs as a result of depressive symptoms or vice versa has implications for how public health practitioners deliver preventive interventions to both LSMY and Latinx non-SMY. Additionally, future research should examine the etiology (i.e., psychological or social mechanisms) of class membership. A tailored approach to family-based preventive interventions can help elucidate the root causes of these outcomes, which can subsequently help identify priority areas for intervention and improve the likelihood of intervention success for LSMY and Latinx non-SMY.

This study has some limitations that should be acknowledged. First, the sample for this study came from a single geographic area, Miami-Dade County, which may not be representative of Latinxs in the United States. Our sample represented a heterogeneous group of Latinxs and LSMY from countries in the Caribbean, Central America, and Latin America, limiting the generalizability of these findings to individuals in other countries and racial/ethnic groups. Second, the data for this study was self-reported by adolescents, and depressive symptoms and drug use may have been underreported or overreported. Relatedly, there may have been Latinx non-SMY who were in fact LSMY but did not answer questions related to sexual orientation due to fears of being “outed” by survey responses [52]. Results may therefore have varied because youth who were in fact LSMY were categorized as Latinx non-SMY. Third, there were no measures that indicated whether LSMY had disclosed their sexual orientation to parents, which limits our understanding of how family functioning may or may not be impacted by the disclosure of an adolescent’s sexual orientation. Similarly, measures of cultural values (e.g., machismo and familism) and family rejection, which are important risk and protective factors for LSMY, were not included in this study. Future studies should assess how these variables impact depressive symptoms and drug use. Fourth, the sample size of LSMY was small (*n* = 46). This highlights the need for continued research with LSMY to draw informed conclusions and attain scientific equity in the amount of knowledge produced for marginalized and overlooked populations such as LSMY [53]. Relatedly, one of the classes had a sample size of 5.9%; however, as noted by Nylund-Gibson and Choi [40], sample sizes as small as 30 may be sufficient for LCA, and our fit indices indicated good fit to the data. Relatedly, the small sample may limit the generalizability of the findings to this particular subgroup. Finally, although this study examined drug use and depressive symptom trajectories over two years, we were unable to ascertain which occurred first or possible confounders to these patterns and relationships. It may be that at some point in the two-year period, youth became engaged in mental healthcare, initiated substance use, or there were changes in family relationships that were not accounted for in this analysis.

## 5. Conclusions

Using person-centered methods and sub-group analysis, this study was able to provide insights related to the heterogeneity of depressive symptoms and drug use among LSMY and Latinx non-SMY. Identification of such unobserved sub-groups can further inform tailored preventive interventions to ameliorate health disparities related to depressive symptoms and drug use.

## Figures and Tables

**Table 1:** Comparison of sociodemographic characteristics and study variables.

	Overall	LSMY	Latinx Non-SMY	*p*-Value ^1,2^
	*n* = 231	*n* = 46	*n* = 169	
Age M (SD)	*n* = 231	*n* = 46	*n* = 169	
	13.84 (1.38)	13.83 (1.37)	13.85 (1.37)	0.934
Percent Female N (%)	*n* = 231	*n* = 46	*n* = 169	
	119 (51.5%)	36 (78.3%)	72 (42.6%)	**<0.001**
Percent Born in the US N (%)	*n* = 231	*n* = 46	*n* = 169	
	128 (55.4%)	24 (52.2%)	92 (54.4%)	0.785
Percent in US 10 years or more N (%)	*n* = 221	*n* = 46	*n* = 159	
	137 (59.3%)	29 (63.0%)	95 (59.7%)	0.768
Frequency of Past 90-day Drug Use M (SD)	*n* = 220	*n* = 46	*n* = 164	
	0.50 (4.33)	0.46 (1.82)	0.54 (4.92)	0.762
Depressive Symptoms M (SD)	*n* = 213	*n* = 45	*n* = 161	
	14.26 (11.32)	20.93 (13.89)	12.11 (9.35)	**<0.001**
Parent-Adolescent Communication M (SD)	*n* = 212	*n* = 45	*n* = 158	
	71.59 (13.19)	68.31 (12.98)	72.83 (13.37)	**0.045**
Parental Monitoring of Peers M (SD)	*n* = 222	*n* = 45	*n* = 165	
	9.43 (4.48)	9.41 (4.80)	9.56 (4.48)	0.424
Positive Parenting M (SD)	*n* = 219	*n* = 44	*n* = 166	
	22.57 (6.54)	21.51 (6.06)	22.92 (6.70)	0.092
Parental Involvement M (SD)	*n* = 214	*n* = 42	*n* = 163	
	42.06 (9.34)	41.05 (8.58)	42.29 (9.64)	0.213
Family Communication M (SD)	*n* = 213	*n* = 44	*n* = 158	
	6.31 (1.83)	5.91 (1.89)	6.45 (1.77)	0.094

Note: Totals vary across variables due to item non-response. Bold *p*-value represents significant value. ^1^ *p*-values derived from independent sample *t*-tests comparing LSMY to Latinx non-SMY. ^2^ *p*-values from Pearson chi-square analysis comparing LSMY to Latinx non-SMY.

**Table 2:** Fit indices for LCA models for the overall sample, LSMY, and Latinx non-SMY.

	Class Size (n, %)	BIC	SSBIC	Entropy	Adj. LMR-LRT (*p* Value)
Overall Sample					
2 Class	(149, 64.8%), (81, 35.2%)	6818.680	6771.139	0.727	539.750 (0.0859)
3 Class	(125, 54.3%), (23, 10.0%), (82, 35.6%)	6664.407	6601.019	0.748	175.026 (0.0708)
4 Class	(19, 8.3%), (19, 8.3%), (69, 30.0%), (123, 53.5%)	6580.208	6500.973	0.795	107.437 (0.0272)
LSMY					
2 Class	(22, 47.8%), (24, 52.1%)	1504.557	1457.525	0.788	157.063 (0.0191)
3 Class	(21, 45.6%), (10, 21.7%), (15, 32.6%)	1481.040	1418.330	0.836	40.543 (0.0095)
4 Class	(9, 19.6%), (16, 34.8%), (13, 28.3%), (8, 17.4%)	1477.961	1399.573	0.810	21.120 (0.0169)
Latinx non-SMY					
2 Class	(127, 75.1%), (42, 24.9%)	4806.714	4759.219	0.761	376.876 (0.004)
3 Class	(99, 58.6%), (60, 35.5%), (10, 5.9%)	4718.264	4654.938	0.778	109.818 (0.1940)
4 Class	(11, 6.5%), (8, 4.7%), (55, 32.5%), (95, 56.2%)	4710.168	4631.011	0.805	32.479 (0.4113)

**Table 3:** Known class analysis model parameter comparisons.

Parameter	χ^2^	df	*p*-Value
Initial Levels of Depressive Symptoms			
Class 1	6.839	1	**0.0089**
Class 2	35.130	1	**<0.001**
Class 3	7.299	1	**0.0069**
Slope Levels of Depressive Symptoms			
Class 1	0.386	1	0.5342
Class 2	7.236	1	**0.0071**
Class 3	1.989	1	0.1585
Initial Levels of Drug Use			
Class 1	13.082	1	**0.0003**
Class 2	1.195	1	0.2744
Class 3	602.148	1	**<0.001**
Slope Levels of Drug Use			
Class 1	33.779	1	**<0.001**
Class 2	0.000	1	0.9940
Class 3	440.247	1	**<0.001**

Note: Bold *p*-value represents significant value.

**Table 4:** Covariate Effects by Class Membership.

	Latinx Non-SMY			LSMY		
	Class 1: Low Depressive Symptoms with High Drug Use That Decreases over Time	Class 2: Moderate Depressive Symptoms with Moderate but Stable Drug Use	Class 3: High Depressive Symptoms with Low Drug Use That Increases over Time	Class 1: Increasing Depressive Symptoms with Moderate Drug Use That Is Stable over Time	Class 2: High Depressive Symptoms that Decrease over Time with Low Drug Use That Increases over Time	Class 3: Moderate Depressive Symptoms that Decrease over Time with Low Drug Use That Increases over Time
**Adolescent Characteristics**						
Age M (SD)	13.46 (1.33)	14.05 (1.37)	13.77 (1.37)	13.00 (1.15)	13.92 (1.66)	14.13 (1.18)
Gender N (%) ***						
Male	7 (6.5%)	33 (30.8%)	57 (53.3%)	0 (0.0%)	0 (0.0%)	10 (9.3%)
Female	6 (5.6%)	26 (24.1%)	40 (37.0%)	10 (9.3%)	13 (12.0%)	13 (12.0%)
Nativity N (%)						
U.S. Born	9 (7.8%)	30 (25.9%)	53 (45.7%)	5 (4.3%)	6 (5.2%)	13 (11.2%)
Foreign Born	4 (4.0%)	29 (29.3%)	44 (44.4%)	5 (5.1%)	7 (7.1%)	10 (10.1%)
Time in U.S. N (%)						
<3 years	2 (3.5%)	18 (31.6%)	24 (42.1%)	3 (5.3%)	3 (5.3%)	7 (12.3%)
3 to 10 years	1 (4.2%)	7 (29.2%)	12 (50.0%)	1 (4.2%)	2 (8.3%)	1 (4.2%)
More than 10 years	9 (7.3%)	30 (24.2%)	56 (45.2%)	6 (4.8%)	8 (6.5%)	15 (12.1%)
Family Functioning M (SD)						
PAC ***	56.75 (15.05)	70.13 (10.68)	76.87 (12.55)	64.05 (17.05)	63.35 (7.82)	72.76 (12.05)
Parental Monitoring of Peers	8.85 (5.32)	8.50 (4.30)	10.30 (4.37)	11.20 (4.73)	7.69 (4.34)	9.53 (4.93)
Positive Parenting	21.99 (7.19)	22.11 (6.48)	23.53 (6.78)	18.19 (3.11)	23.00 (6.10)	22.24 (6.65)
Parental Involvement	37.42 (11.59)	41.29 (8.53)	43.43 (9.88)	39.79 (7.85)	36.23 (9.11)	43.98 (7.67)
Family Communication	5.42 (2.57)	6.30 (1.62)	6.68 (1.69)	6.00 (2.05)	5.33 (1.83)	6.18 (1.87)
**Family Characteristics**						
Nativity N (%)						
U.S. Born	2 (7.7%)	4 (15.4%)	15 (57.7%)	1 (3.8%)	1 (3.8%)	3 (11.5%)
Foreign Born	11 (5.8%)	55 (29.1%)	82 (43.4%)	9 (4.8%)	12 (6.3%)	20 (10.6%)
Time in U.S. N (%)						
<3 years	2 (3.7%)	19 (35.2%)	23 (42.6%)	3 (5.6%)	3 (5.6%)	4 (7.4%)
3 to 10 years	2 (7.4%)	6 (22.2%)	14 (51.9%)	0 (0.0%)	2 (7.4%)	3 (11.1%)
More than 10 years	9 (6.8%)	33 (24.8%)	60 (45.1%)	7 (5.3%)	8 (6.0%)	16 (12.0%)
Parent Education Level N (%)						
None	0 (0.0%)	0 (0.0%)	0 (0.0%)	0 (0.0%)	0 (0.0%)	1 (100.0%)
Elementary	1 (9.1%)	1 (9.1%)	4 (36.4%)	1 (9.1%)	1 (9.1%)	3 (27.3%)
High School	7 (11.1%)	16 (25.4%)	33 (52.4%)	3 (4.8%)	2 (3.2%)	2 (3.2%)
College	4 (4.0%)	28 (28.3%)	44 (44.4%)	4 (4.0%)	7 (7.1%)	12 (12.1%)
Graduate/Professional School	1 (2.6%)	13 (33.3%)	14 (35.9%)	2 (5.1%)	3 (7.7%)	6 (15.4%)
Family Income N (%)						
<$30,000	7 (6.4%)	24 (22.0%)	53 (48.6%)	6 (5.5%)	8 (7.3%)	11 (10.1%)
Between $30,000–$50,000	1 (4.0%)	8 (32.0%)	11 (44.0%)	0 (0.0%)	2 (8.0%)	3 (12.0%)
>$50,000	1 (3.4%)	11 (37.9%)	11 (37.9%)	2 (6.9%)	2 (6.9%)	2 (6.9%)

Note: Totals vary across variables due to item non-response. *** *p* < 0.001.

## Data Availability

No new data were created or analyzed in this study. Data sharing is not applicable to this article.

## Supplementary Materials

The following supporting information can be downloaded at: https://www.mdpi.com/article/10.3390/ijerph20105883/s1, Table S1: Tukey HSD Multiple Comparisons for Parent-Adolescent Communication.
