# Supplementary material for: Trajectories of Drug Use and Depressive Symptoms among Latinx Youth and Sexual Minority Youth
Source: Int J Environ Res Public Health. 2023 May 19;20(10):5883. doi: 10.3390/ijerph20105883 (PMC10218419; doi:10.3390/ijerph20105883)
Supplement: Supplementary file 1 [file ijerph-20-05883-s001.zip › ijerph-2356571-supplementary.pdf]

Supplemental Table S1

Tukey HSD Multiple Comparisons for Parent-Adolescent Communication

Class	Comparison Class	Mean Difference	Std. Error	*p*-value	95% Confidence Interval
**Latina/o/x non-SMY Classes**					
Class 1 non-LSMY	Class 2 non-LSMY	-13.38	3.76	**0.006**	[-24.19, -2.57]
	Class 3 non-LSMY	-20.11	3.62	**<0.001**	[-30.54, -9.69]
	Class 1 LSMY	-7.29	5.13	0.714	[-22.06, 7.48]
	Class 2 LSMY	-6.60	4.88	0.756	[-20.65, 7.46]
	Class 3 LSMY	-16.00	4.23	**0.003**	[-28.18, -3.82]
Class 2 non-LSMY	Class 1 non-LSMY	13.38	3.76	**0.006**	[2.57, 24.19]
	Class 3 non-LSMY	-6.73	2.08	**0.018**	[-12.72, -0.74]
	Class 1 LSMY	6.09	4.19	0.694	[-5.97, 18.14]
	Class 2 LSMY	6.79	3.88	0.501	[-4.38, 17.96]
	Class 3 LSMY	-2.62	3.02	0.954	[-11.32, 6.08]
Class 3 non-LSMY	Class 1 non-LSMY	20.11	3.62	**<0.001**	[9.69, 30.54]
	Class 2 non-LSMY	6.73	2.08	**0.018**	[0.74, 12.72]
	Class 1 LSMY	12.82	4.07	**0.023**	[1.11, 24.53]
	Class 2 LSMY	13.52	3.75	**0.005**	[2.72, 24.32]
	Class 3 LSMY	4.11	2.85	0.703	[-4.10, 12.32]
**LSMY Classes**					
Class 1 LSMY	Class 1 non-LSMY	7.29	5.13	0.714	[-7.48, 22.06]
	Class 2 non-LSMY	-6.09	4.19	0.694	[-18.14, 5.97]
	Class 3 non-LSMY	-12.82	4.07	**0.023**	[-24.53, -1.11]
	Class 2 LSMY	0.70	5.22	1	[-14.34, 15.73]
	Class 3 LSMY	-8.71	4.62	0.415	[-22.01, 4.59]
Class 2 LSMY	Class 1 non-LSMY	6.60	4.88	0.756	[-7.46, 20.65]
	Class 2 non-LSMY	-6.79	3.88	0.501	[-17.96, 4.38]
	Class 3 non-LSMY	-13.52	3.75	**0.005**	[-24.32, -2.72]
	Class 1 LSMY	-0.70	5.22	1	[-15.73, 14.34]
	Class 3 LSMY	-9.41	4.34	0.259	[-21.91. 3.10]
Class 3 LSMY	Class 1 non-LSMY	16.00	4.23	**0.003**	[3.82, 28.19]
	Class 2 non-LSMY	2.62	3.02	0.954	[-6.08, 11.32]
	Class 3 non-LSMY	-4.11	2.85	0.703	[-12.32, 4.10]
	Class 1 LSMY	8.71	4.62	0.415	[-4.59, 22.01]
	Class 2 LSMY	9.41	4.34	0.259	[-3.10, 21.91]
